# Accumulation of Pathological Prion Protein PrP^Sc^ in the Skin of Animals with Experimental and Natural Scrapie

**DOI:** 10.1371/journal.ppat.0030066

**Published:** 2007-05-25

**Authors:** Achim Thomzig, Walter Schulz-Schaeffer, Arne Wrede, Wilhelm Wemheuer, Bertram Brenig, Christine Kratzel, Karin Lemmer, Michael Beekes

**Affiliations:** 1 P24 Transmissible Spongiform Encephalopathies, Robert Koch-Institut, Berlin, Germany; 2 Prion and Dementia Research Unit, Department of Neuropathology, Universitätsklinikum Göttingen, Göttingen, Germany; 3 Institute of Veterinary Medicine, Georg-August-Universität Göttingen, Göttingen, Germany; Institute for Animal Health, United Kingdom

## Abstract

Prion infectivity and its molecular marker, the pathological prion protein PrP^Sc^, accumulate in the central nervous system and often also in lymphoid tissue of animals or humans affected by transmissible spongiform encephalopathies. Recently, PrP^Sc^ was found in tissues previously considered not to be invaded by prions (e.g., skeletal muscles). Here, we address the question of whether prions target the skin and show widespread PrP^Sc^ deposition in this organ in hamsters perorally or parenterally challenged with scrapie. In hamsters fed with scrapie, PrP^Sc^ was detected before the onset of symptoms, but the bulk of skin-associated PrP^Sc^ accumulated in the clinical phase. PrP^Sc^ was localized in nerve fibres within the skin but not in keratinocytes, and the deposition of PrP^Sc^ in skin showed no dependence from the route of infection and lymphotropic dissemination. The data indicated a neurally mediated centrifugal spread of prions to the skin. Furthermore, in a follow-up study, we examined sheep naturally infected with scrapie and detected PrP^Sc^ by Western blotting in skin samples from two out of five animals. Our findings point to the skin as a potential reservoir of prions, which should be further investigated in relation to disease transmission.

## Author Summary

Transmissible spongiform encephalopathies (TSEs), or prion diseases, are fatal neurodegenerative diseases affecting the central nervous system. According to the prion hypothesis, TSEs are caused by proteinaceous infectious particles (“prions”) that consist essentially of PrP^Sc^, an aberrant form of the prion protein with a pathologically altered folding and/or aggregation structure. Scrapie of sheep, chronic wasting disease (CWD) of deer, bovine spongiform encephalopathy (BSE) of cattle, and variant Creutzfeldt-Jakob disease (vCJD) of humans are prominent examples of acquired prion diseases. To further pinpoint the peripheral tissues that could serve as reservoirs of prions in the mammalian body and from which these pathogens could be potentially disseminated into the environment and transmitted to other individuals, we examined the skin of hamsters perorally challenged with scrapie and of naturally infected scrapie sheep for the presence of PrP^Sc^. We show that PrP^Sc^ can accumulate in the skin at late stages of incubation, and that the protein is located primarily in small nerve fibres within this organ. The question of whether the skin may also provide a reservoir for prions in CWD, BSE, or vCJD, and the role of the skin in relation to the natural transmission of scrapie in the field needs further investigation.

## Introduction

Transmissible spongiform encephalopathies (TSEs), or prion diseases, are fatal neurodegenerative diseases affecting both animals and humans. According to the prion hypothesis, TSEs are caused by infectious prions that consist essentially—if not entirely— of a misfolded form of the prion protein (PrP), which is known as PrP^Sc^ [1]. Although the precise molecular composition and structure of prions remains elusive, PrP^Sc^ has been shown in many studies to accumulate together with infectivity in target tissues of infection and is therefore considered a reliable biochemical marker for TSE agents [2] as reported for experimentally challenged hamsters [3], other animal species [4], and humans [5,6].

Scrapie of sheep and goats, chronic wasting disease (CWD) of deer, bovine spongiform encephalopathy (BSE) of cattle, and variant Creutzfeldt-Jakob disease (vCJD) of humans represent acquired prion diseases that are caused by exposure to TSE agents in the living environment of the respective host. Different lines of evidence suggest that many, if not the majority, of cases of ovine scrapie, BSE, and purportedly CWD are caused by ingestion of prions and subsequent invasion of the organism via the alimentary tract [7]. This also holds true for vCJD, which is now generally acknowledged to be acquired through consumption of BSE-contaminated foodstuffs [8].

Although the exact mechanism of infection following passage of prions through the alimentary tract has not yet been completely elucidated, findings from different mammalian species suggested that the infection ascended retrogradually via peripheral nerves to the spinal cord and to the brain (for reviews see [2,7]). From these sites of initial central nervous system invasion at the level of the thoracic spinal cord and the medulla oblongata, the infection propagated in both ascending and descending directions [2,3,7,9–11]. Centrifugal spread from the central nervous system appeared to be responsible for subsequent infection of further parts of the peripheral nervous system [9,11]. In particular, PrP^Sc^ was found associated with nerve fibres or nerve endings innervating peripheral organs and tissues such as muscles [11–14]. This prompted us to look for further tissues which could serve as reservoirs of prions in the mammalian body, and from which these pathogens could be potentially disseminated into the environment and transmitted to other individuals via peroral or alternative routes. In this context, the skin appears to be of utmost importance. The skin consists of different strata and appendages which are highly innervated and interspersed with lymphatics and blood vessels [15]. It constitutes the largest organ of humans and many animal species and provides an interface with their environment. However, although PrP^Sc^ detection has been reported for mucosal tissue [16,17], the skin has not been extensively studied for the presence of prions and PrP^Sc^ so far. In 2004, Cunningham et al. reported on the presence of BSE agent in a wide range of tissues from a BSE-infected greater kudu [18]. In one animal of this study, the salivary gland and skin were found to contain infectivity, and the authors suggested that these findings possibly indicate routes by which direct animal-to-animal transmission of the disease may occur.

Here, we examined the skin of prion-infected hamsters for the presence of PrP^Sc^. Our hamster experiments focussed on orally infected animals, which have been previously established as a relevant rodent model to study the spread of prions in the peripheral nervous system [2,7]. These studies were performed in order to (i) investigate whether anatomical structures within the skin may provide a target for PrP^Sc^ accumulation, (ii) elucidate the identity of such skin components, and (iii) find out whether prions can be present in the skin prior to the onset of visible TSE symptoms. In a proof-of-concept approach, we extended PrP^Sc^ testing of the skin to specimens from sheep naturally infected with scrapie. This follow-up study intended to obtain further insights into the pathophysiology of scrapie and the putative pathways of its natural transmission in the field.

## Results

### PrP^Sc^ Accumulation in the Skin following Peroral Infection with Scrapie Becomes Detectable Shortly before the Onset of Clinical Symptoms

To investigate whether and at which stages of scrapie infection PrP^Sc^ accumulates in the skin, we performed a time-course study in hamsters orally exposed to 263K scrapie agent. As established previously for the examination of muscle tissue [11], PrP^Sc^ was visualized by sensitive Western blotting after extraction of the protein in the form of its protease-resistant core, PrP27–30, from skin specimens by a high-yield purification method.

We examined samples of skin tissue from hamsters taken at five different time points, i.e., at 70, 100, and 130 days post infection (dpi) in the pre-clinical phase of incubation, at the onset of clinical symptoms, and at the end stage of disease, which occurred after 164 ± 6 d (expressed as the mean ± standard deviation [SD]; *n* = 5 ). Skin samples from the following five body regions of each hamster were analyzed: the forelimb, the hindlimb, the abdomen, the back, and the head.

**Figure 1:**
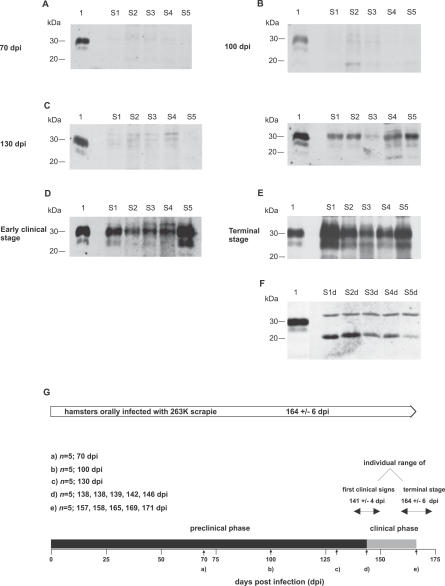
Time-Course of PrP^Sc^ Deposition in Skin Tissue (A–E) Western blot detection of PrP27–30, the protease-resistant core of PrP^Sc^, extracted from different skin samples of hamsters orally challenged with 263K scrapie and sacrificed at the following time-points after infection: (A) 70 dpi, (B) 100 dpi, (C) 130 dpi, (D) at the onset of clinical signs for scrapie (138–146 dpi), and (E) at the terminal stage of disease (157–171 dpi). Lanes with test samples: S1, skin sample from hindlimb; S2, skin sample from forelimb; S3, skin sample from back; S4, skin sample from abdomen; S5, skin sample from head. Lanes with control samples: 1, proteinase K–digested brain homogenate from terminally ill 263K scrapie hamsters containing 1 × 10^−7^ g brain tissue. Representative results are shown for each stage of incubation. Substantial individual variation was observed at 130 dpi, with two of five and three of five animals displaying findings as in (C) in the Western blot on the left-hand side or the Western blot on the right-hand side, respectively. (F) Lanes S1d–S5d: Same samples as in S1–S5 of (E) but deglycosylated with PNGaseF. (A–F) Amounts of tissue represented in lanes: (A) S1, 43 mg; S2, 52 mg; S3, 68 mg; S4, 58 mg; S5, 73 mg; (B) S1, 78 mg; S2, 44 mg; S3, 63 mg; S4, 67 mg; S5, 50 mg; ([C], Western blot on the left side) S1, 42 mg; S2, 76 mg; S3, 61 mg; S4, 58 mg; S5, 73 mg; ([C], Western blot on the right side) S1, 51 mg; S2, 63 mg; S3, 70 mg; S4, 87 mg; S5, 54 mg; (D) S1, 63 mg; S2, 68 mg; S3, 90 mg; S4, 50 mg; S5, 68 mg; (E) S1, 55 mg; S2, 73 mg; S3, 80 mg; S4, 88 mg; S5, 70 mg; (F) S1d, 12 mg; S2d, 14 mg; S3d, 19 mg; S4d, 12 mg; S5d, 20 mg. (G) Time-scale displaying the mean incubation period and the pre-clinical and clinical phases of incubation of hamsters orally infected with 263K scrapie. Small vertical arrows indicate time-points at which animals were tested for PrP^Sc^ in skin samples.

PrP27–30 could not be detected in any of the examined skin samples at 70 and 100 dpi from five animals each (Figure 1A and 1B). The earliest unambiguous signals for accumulation of the pathological prion protein PrP^Sc^ were found in skin samples from three out of five animals at 130 dpi in the late pre-clinical phase of incubation, corresponding to about 80% of the mean incubation period until terminal disease (Figure 1C, Western blot on the right-hand side, lanes S1, S2, S4, and S5). However, variable combinations of PrP^Sc^-positive skin samples from different regions of the body were found at 130 dpi, indicating individual variation in the spread of infection to, or inhomogeneous distribution of PrP^Sc^ in skin tissue. Possibly, prion infection of the skin could have been detected more frequently in animals at 130 dpi, or at earlier pre-clinical stages of incubation, by using alternative methodologies such as the conformation-dependent immunoassay [19] or bioassays. At the onset of clinical symptoms, all of the analyzed skin specimens from all five examined hamsters displayed more or less strong signals for PrP27–30 (Figure 1D). At the terminal stage of scrapie, the positive signals for PrP27–30 become more intense, suggesting that accumulation of PrP^Sc^ takes place predominantly in relatively late stages of incubation (Figure 1E, lanes S1–S5). The weight of the tested skin samples ranged from approximately 40 to 100 mg as specified in the legend to Figure 1A–1E. In order to verify that the detected bands originated from PrP^Sc^, a control experiment was performed: After deglycosylation with PNGaseF, the PrP27–30 bands showed an electrophoretic shift towards a single band at about 20 kDa, the molecular weight to be expected for the unglycosylated PrP27–30 form of 263K hamster scrapie (Figure 1F, lanes S1d–S5d). Control samples from mock-challenged age-matched hamsters consistently produced negative results (not shown). A time-scale displaying an overview of the time-points at which the p.o.-infected hamsters were tested for skin-associated PrP^Sc^ deposition in relation to the mean incubation period and the pre-clinical and clincal phases of incubation is provided in Figure 1G.

### Location of PrP^Sc^ within the Skin

**Figure 2:**
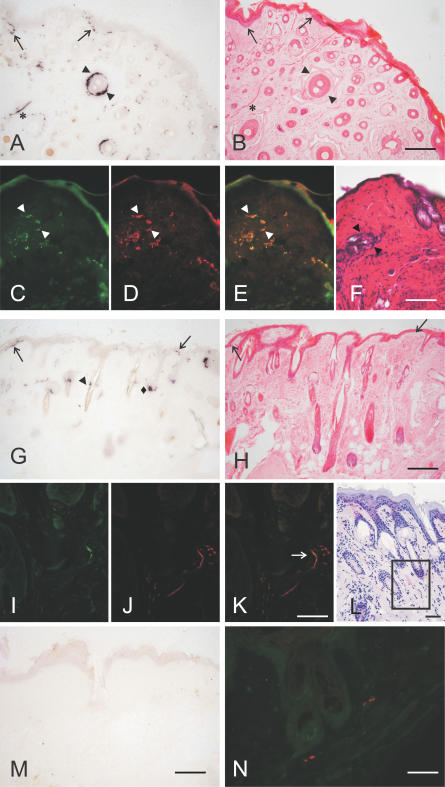
Location of PrP^Sc^ within the Skin of Hamsters Orally Infected with Scrapie (A–H) Topographical localisation of PrP^Sc^ in sections of skin samples from the snout (A and B) and the forelimb (G and H); (A and G) PET blots, (B and H) H&E staining. PrP^Sc^ was detected in free nerve endings of the subepidermal plexus on the border of the epidermis to the dermis ([A], [B], [G], and [H], arrows), in fibres of the subepidermal, the deep cutaneous and the subcutaneous plexus, in circular and longitudinal fibres of the follicular neural network of the hair ([A], [B], and [G], arrowheads), in the hair follicle isthmus ([G]; rhombus), and in small intradermal striated fibres of mimic muscles ([A and B], asterisks). (C–F) Visualisation of PrP^Sc^ and nerve fibres in the neural network of hair follicles by fluorescence microscopy (skin sample from the abdomen). Co-localisation of PrP^Sc^ (C) with nerve fibres labelled by using an anti–S-100 protein antibody against Schwann cells (D). (E) Merged figure from micrographs (C and D). (F) Adjacent section to (C), stained with H&E. (I–K) Visualisation of PrP^Sc^ and nerve fibres in the cutaneous plexus by fluorescence microscopy (skin sample form the snout). Co-localisation of PrP^Sc^ (I) with nerve fibres labelled by using the anti-neurofilament antibody SMI 31 (J). (K) Merged figure from micrographs (I and J). (L) Adjacent section to (I), stained with H&E. The box indicates the region used for the immunofluorescence stainings in (I–K). (M and N) Control skin samples from the forelimb of a hamster perorally mock-challenged with normal hamster brain homogenate; PET blot (M) and fluorescence microscopy for PrP and neurofilament (N). Scale bars = 200 μm for (B, F, H, and M), 50 μm for (K and L), and 25μm for (N). Same scale bars as displayed in (B), (F), (H), and (K) apply to (A), (C–E), (G), and (I and J), respectively.

To determine where in the skin PrP^Sc^ accumulates, we investigated samples from the head, snout, forelimb, and abdomen of orally 263K scrapie–infected, terminally ill hamsters. As done previously when determining the route of PrP^Sc^ propagation to muscles [11], we used the paraffin-embedded tissue (PET) blot method to achieve a sensitive topographical localisation of disease-associated PrP in the skin. Using either Carnoy- or paraformaldehyde-fixed tissue samples, PrP^Sc^ was detectable in (i) free nerve endings of the subepidermal plexus on the border of the epidermis to the dermis (Figure 2A, 2B, 2G, and 2H, arrows), (ii) fibres of the subepidermal, the deep cutaneous, and the subcutaneous plexus, (iii) fibres of the follicular neural network of the hair (circular and longitudinal fibres, Figure 2A, 2B, and 2G, arrowheads), (iv) the hair follicle isthmus (Figure 2G, rhombus), and (v) small intradermal striated fibres of mimic muscles (Figure 2A and 2B, asterisks). No PrP^Sc^ was detectable in keratinocytes, epidermal basal cells, fibroblasts of the dermal connective tissue, capillary blood vessels, outer root sheet cells of the hair, or the bulge region and the sebaceous gland, but PrP^Sc^ was present in nerve fibres of the sebaceous gland (not shown). Nerve fibres in the skin can be labelled by antibodies binding to neurofilament or Schwann cells [20,21]. By using an anti–S-100 protein antibody detecting Schwann cells, a co-localisation of PrP^Sc^ with nerve fibres of the follicular neuronal network of hairs was observed (Figure 2C–2E, arrowheads). By antibody-labelling of neurofilament, nerve fibres of the cutaneous plexus were found to display a co-localisation with PrP^Sc^ (Figure 2I–2K; for topological orientation, see Figure 2L). Occasionally, we observed PrP^Sc^ deposition in nerve-like structures of the subepidermal plexus showing no immunostaining for neurofilament (not shown). This may have resulted from infection of nerve fibres that do not contain neurofilament [20], or, alternatively, from PrP^Sc^ deposition in Schwann cells rather than in the neurite itself [22]. Control skin samples from the forelimb of a hamster perorally mock-challenged with normal hamster brain homogenate were found negative for PrP^Sc^ by PET blotting and fluorescence microscopy (Figure 2M and 2N).

### Accumulation of PrP^Sc^ in the Skin Shows No Dependence on the Route of Infection and Lymphotropic Spread

**Figure 3:**
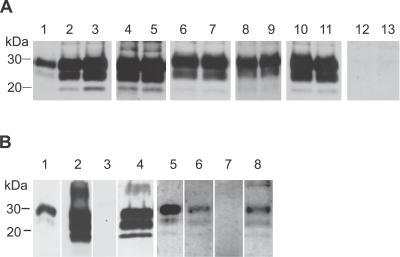
PrP^Sc^ Routing to the Skin and to Components of the Lymphoreticular System of Hamsters Challenged via Different Routes with 263K Scrapie Agent (A) Western blot detection of PrP27–30, the protease-resistant core of PrP^Sc^, in skin specimens from terminally ill scrapie hamsters. Lanes 1, 2, and 3: skin samples from orally mock-infected control hamsters, spiked before extraction with 1 × 10^−6^ g, 5 × 10^−6^ g, or 1 × 10^−5^ g of brain homogenate from terminally ill 263K hamsters. Lanes 4 and 5: skin samples from hindlimbs and forelimbs of hamsters orally infected with scrapie brain homogenate. Lanes 6 and 7: skin samples from hindlimbs and forelimbs of hamsters intracerebrally infected with scrapie brain homogenate. Lanes 8 and 9: skin samples from hindlimbs and forelimbs of hamsters infected by implantation of s.w. contaminated with scrapie agent. Lanes 10 and 11: skin samples from hindlimbs and forelimbs of hamsters infected peripherally by f.p. inoculation of scrapie brain homogenate. Lanes 12 and 13: skin samples from hindlimbs and forelimbs of hamsters orally mock-infected with normal brain homogenate. Amounts of tissue represented in lanes: 1, 53mg; 2, 58 mg; 3, 68 mg; 4, 68 mg; 5, 75 mg; 6, 78 mg; 7, 64 mg; 8, 69 mg; 9, 60 mg; 10, 62 mg; 11, 73 mg; 12, 61 mg; 13, 58 mg. (B) Western blot detection of PrP27–30 in spleens and selected lymph nodes from terminally ill scrapie hamsters. Lanes 1 and 5: proteinase K-digested brain homogenate from terminally ill scrapie hamsters, containing 1 × 10^−7^ g brain tissue. Lanes 2–4: spleen samples from p.o.- (2), s.w.-, (3) and i.c.-infected (4) hamsters. Lanes 6–8: mesenteric lymph node samples from p.o.- (6), s.w.-, (7) and i.c.-infected (8) hamsters. Amounts of tissue represented in lanes: 2, 40 mg; 3, 45 mg; 4, 41 mg; 6, 6 mg; 7, 8 mg; 8, 6 mg.

In order to examine whether accumulation of PrP^Sc^ in the skin depends on the mode of infection and spreading pathways other than the nervous system, skin specimens from the forelimb and hindlimb of terminally ill hamsters challenged with 263K scrapie agent by different routes were analyzed using the same analytical approach as described above for the time-course study. The following modes of inoculation were compared: oral (p.o.) infection with scrapie brain homogenate (Figure 3A, lanes 4 and 5), intracerebral (i.c.) infection with scrapie brain homogenate (Figure 3A, lanes 6 and 7), i.c. infection by implantation of steel wires (s.w.) contaminated with scrapie agent (Figure 3A, lanes 8 and 9), and peripheral foot pad (f.p.) infection with scrapie brain homogenate (Figure 3A, lanes 10 and 11). PrP27–30 could be detected in all analyzed skin specimens from hamsters at the terminal stage of scrapie independently of the route of infection. Amounts of PrP^Sc^ in skin tissue were about 5,000- to 10,000-fold lower than those found in brain, as estimated from positive controls of skin samples from orally mock-infected hamsters that were spiked with 1 × 10^−6^ g, 5 × 10^−6^ g, or 1 × 10^−5^ g of homogenized 263K scrapie hamster brain from terminally ill donors before extraction (Figure 3A, lanes 1–3). PrP^Sc^ was consistently absent in skin specimens from mock-infected hamsters, which served as negative controls (Figure 3A, lanes 12 and 13).

The employed routes of inoculation were found to produce substantial differences in the extent of lymphotropic spread of agent. In orally or intracerebrally challenged hamsters, spleens (Figure 3B, lanes 2 and 4), mesenterial lymph nodes (Figure 3B, lanes 6 and 8) and retropharyngeal lymph nodes (not shown) consistently showed PrP^Sc^ deposition, whereas in five out of six hamsters infected by implantation of contaminated s.w., no PrP^Sc^ could be found in the examined spleens and lymph node specimens (Figure 3B, lanes 3 and 7). Only one of the s.w.-infected animals displayed a weak Western blot signal for PrP^Sc^ in the spleen (not shown). Thus, in comparison to the i.c. or p.o. route of inoculation, lymphotropic spread was much less pronounced—or even undetectable—following infection of hamsters by i.c. implantation of 263K-contaminated s.w. However, despite this marked discrepancy, the intensity of skin-associated deposits of PrP^Sc^ found in s.w.-infected hamsters did not show significant differences from that observed for p.o.- or i.c.-challenged animals upon Western blotting (Figure 3A, lines 8 and 9 versus lines 4–7). To determine whether the route of administration of PrP^Sc^ influences the topographical distribution of disease-associated prion protein in the skin, we investigated samples from hamsters infected with 263K-contaminated s.w. at the clinical disease stage using the PET blot method. This revealed that the morphological distribution pattern of PrP^Sc^ in the skin of s.w.-infected animals was identical to that found in orally infected hamsters (not shown).

### BSE-Associated PrP^Sc^ in the Skin

**Figure 4:**
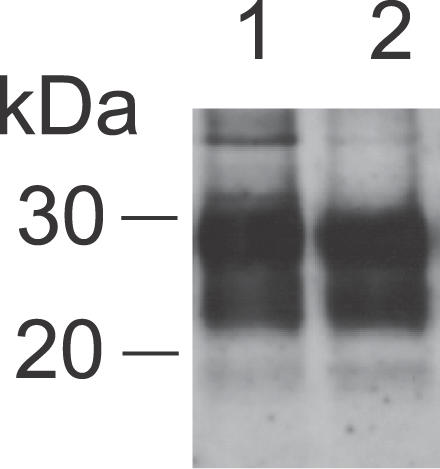
Western Blot Detection of PrP27–30 in Skin Specimens of Hamsters Intracerebrally Challenged with BSE-H Agent Lanes 1 and 2: skin samples from hindlimb and forelimb of a BSE-infected hamster. Amounts of tissue represented in lanes: 1, 43 mg; 2, 58 mg.

To test whether not only scrapie-associated PrP^Sc^ but also BSE-associated PrP^Sc^ accumulates in the skin, hamsters were intracerebrally infected with a hamster-adapted BSE (BSE-H) agent and sacrificed at the end stage of disease. All analyzed skin specimens from forelimbs and hindlimbs of five donor animals showed substantial amounts of BSE-associated PrP27–30 (Figure 4, lanes 1 and 2). Thus, following i.c. infection, the targeting of BSE-H agent to the skin, as probed by Western blotting, did not show discernible differences compared to that observed for 263K scrapie.

### PrP^Sc^ in Skin Tissue of Sheep Naturally Infected with Scrapie

**Figure 5:**
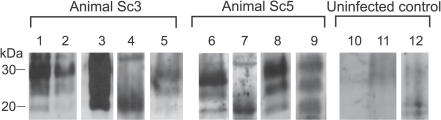
PrP^Sc^ in Skin Samples from Different Body Areas of Sheep Naturally Infected with Scrapie Western blot detection of PrP27–30, the protease-resistant core of PrP^Sc^, using the anti-PrP antibodies ICSM-18 (lanes 1–8, 10–12) and P4 (lane 9). Samples are from sheep Sc3 (lanes 1–5), sheep Sc5 (lanes 6–9), and from an uninfected control sheep. Lanes 1 and 2: different amounts of brain homogenate (0.5 mg/lane and 0.1 mg/lane of midbrain tissue, respectively) from sheep Sc3. Lane 3: tonsil sample. Lane 4: tonsil sample after deglycosylation with PNGaseF. Lane 5: skin sample from the inguinal region. Lane 6: skin sample from the perianal region (scratching area). Lane 7: skin sample from the same region as in lane 6 after deglycosylation with PNGaseF. Lane 8: skin sample from the snout. Lane 9: skin sample from the same region as in lane 8, but detected with the antibody P4. Lanes 10–12: negative controls of skin samples from different body regions of an uninfected sheep. Amounts of tissue represented in lanes: 1, 0.5 mg; 2, 0.1 mg; 3, 7 mg; 4, 2 mg; 5, 88 mg; 6, 89 mg; 7, 23 mg; 8, 90 mg; 9, 92 mg; 10, 80 mg; 11, 87 mg; 12, 94 mg.

In a follow-up proof-of-concept study, which aimed to validate and expand our findings from the hamster experiments, five sheep naturally infected with scrapie in the field were analyzed for the presence of PrP^Sc^ in skin specimens from different body regions (head, snout, hindlimb, forelimb, perianal, axillar, and inguinal). PrP^Sc^ was visualized by Western blotting after extraction in the form of PrP27–30 from tissue samples using the anti-ovine PrP monoclonal antibodies ICSM-18 or P4. Positive specimens were found in two out of the five tested sheep: In sheep Sc3, PrP27–30 was present in a sample from the inguinal region (Figure 5, lane 5). Sheep Sc5 showed PrP27–30 in a sample from the perianal region, which was a scratching site of this animal (Figure 5, lane 6). For control purposes, a deglycosylation of a PrP27–30 extract from the same skin region of this animal was performed (Figure 5, lane 7). Furthermore, a skin sample from the snout was found positive for PrP27–30 in animal Sc5 upon detection with the ICSM-18 antibody (Figure 5, lane 8) and the anti-PrP antibody P4 (Figure 5, lane 9). All other analyzed skin specimens of the five sheep did not show specific Western blot signals for PrP27–30. Specimens from the same skin regions of four uninfected sheep served as controls and consistently produced negative results (Figure 5, lanes 10–12).

## Discussion

In this study we have shown that the skin provides a reservoir for PrP^Sc^, the biochemical marker of prion infectivity, in five different hamster TSE models, independently of whether the animals were challenged with scrapie via the p.o., i.c., or f.p. route, cerebral implantation of scrapie-contaminated s.w., or i.c. inoculation of a hamster-adapted BSE agent. Furthermore, PrP^Sc^ could be demonstrated for the first time in skin specimens from sheep naturally infected with scrapie, though in a limited number of sites investigated and at low amounts. In a time-course study using hamsters fed with scrapie agent, we were able to detect PrP^Sc^ in the skin before the onset of clinical symptoms, but the bulk of skin-associated PrP^Sc^ accumulated in the clinical phase of the disease. From our Western blot findings, the final concentration of PrP^Sc^ in the skin of hamsters seems to be approximately 5,000–10,000 times lower than that found in the brain. This would correspond to an infectivity titre of ~ 1 × 10^5^ to 2 × 10^5^ 50% i.c. infective doses (ID_50i.c._) per gram of skin tissue. A similar infectivity titre was previously estimated from Western blot findings for skeletal muscle tissue of clinically ill hamsters perorally challenged with 263K scrapie [23].

### Pathophysiology of PrP^Sc^ Deposition in the Skin

Our immunohistochemical and PET blot studies, performed on skin specimens from hamsters orally infected with scrapie in order to elucidate the topographical location of PrP^Sc^ in the skin, revealed PrP^Sc^ in small nerve fibres within the dermis but not in keratinocytes. Keratinocytes have been shown to express PrP^C^ [24,25], and it remains to be established in future studies why this cell type—other than, for example, myocytes—does not support a detectable formation of PrP^Sc^. Irrespective of this uncertainty, the topographical dermal location of PrP^Sc^, which is essentially restricted to neural structures together with the late occurrence of the PrP^Sc^ in the skin, point to an invasion via centrifugal spread of infection along peripheral nerves. The time-course of PrP^Sc^ accumulation in the skin, and the putative neural spreading pathways used by prions to target this organ, are strongly reminiscent of what has been previously observed for muscle tissue in the same animal model [11]. In order to further examine whether pathways other than the nervous system may be involved in the spread of prions to the skin, a group of hamsters was intracerebrally infected by implantation of s.w. contaminated with scrapie agent. In this experimental paradigm, lymphotropic spread of scrapie agent through the body, as evidenced by testing the spleen and selected lymph nodes for PrP^Sc^, was not detectable in five out of six animals. Only in the spleen of one hamster from the s.w. group was a weak signal for PrP^Sc^ found. This is indicative of a splenic infection, which occurred only relatively late in the incubation period, possibly via the peripheral nervous system. Despite the striking absence of detectable lymphotropic spread in s.w.-infected hamsters, these animals produced a practically indistinguishable skin-associated accumulation of PrP^Sc^ from that observed for p.o.- or i.c.-challenged hamsters. Thus, propagation of infection to the skin did not show a crucial dependence on lymphotropic spreading pathways. Whether a blood-borne dissemination of agent contributes to the PrP^Sc^ contamination of skin additonally to neurally mediated invasion remains to be established.

### Skin-Associated PrP^Sc^ Deposition in Sheep Naturally Infected with Scrapie

The Western blot examination of skin specimens from five sheep clinically affected with natural scrapie in the field revealed the presence of PrP^Sc^ in a sample from the inguinal region of one animal, and in two samples from the snout and perianal region of another sheep. The perianal region was a scratching site of this animal but did not show macroscopically visible skin alterations. Although the exact anatomical location of skin-associated PrP^Sc^ in ovine scrapie remains to be determined, our findings clearly demonstrate that the skin of sheep naturally infeceted with scrapie can provide a reservoir for prions at least at late stages of incubation. The results with skin samples from scrapie-infected sheep are in good accordance with the Western blot findings in hamsters, and the anatomical organisation of hamster and sheep skin shows considerable similarities. However, because of possible anatomical differences, histological findings in hamsters cannot be extended directly to sheep without further immunohistochemical and/or PET blot examinations in ovines. Also, for a more precise assessment of potential risks possibly emanating from prions in the skin of scrapie-infected sheep, multiple tissue sites and larger numbers of animals, including those pre-clinically incubating the disease, and random case control studies are required.

Our findings raise the question of whether prions present in specific components of the skin such as peripheral nerve fibres may be involved in the natural transmission of scrapie. Dissemination of infectivity from the skin into the environment could theoretically take place at skin lesions such as scrapie-induced chafing sites and other wounds or ulcers, and vectors found to be able to harbour infectivity (e.g., mites [26] or larvae and pupae [27]) may possibly take up prions from the skin of affected sheep. In this context, it has to be noted that inflammatory processes [25,28] may enhance the load of prions in infected skin regions, and that an increase of cellular prion protein expression was observed in keratinocytes of human patients with inflammatory skin diseases [24]. Furthermore, practises of sheep shearing may account for transmission of scrapie from reservoirs in the skin. Shearing causes skin wounds in up to a third of the sheared animals, and the skin may not only provide a reservoir of prions but also an efficient portal of entry for scrapie agent into the body [29,30]. However, previous studies on the presence of infectivity in which various tissues from scrapie-infected sheep or goats were tested did not point to the skin as a highly relevant reservoir of prions in small ruminants [31]. Furthermore, a substantial body of evidence suggests that many, if not the majority of cases of ovine scrapie, are caused by peroral uptake of TSE agents and subsequent invasion of the organism via the alimentary tract [32–35]. Contaminated placenta [31,36], saliva (as recently reported in the context of CWD) [37], or possibly feces or urine [38] appear to provide more relevant candidate sources of infection than the skin in the horizontal or vertical transmission of scrapie. Thus, whether and to what degree the skin plays a role in the spread of contagious scrapie in the field, and whether BSE-infected cattle, CWD-infected deer or vCJD-infected humans may also accumulate PrP^Sc^ in the skin, remains to be established in future studies.

## Materials and Methods

### Experiments in laboratory animals.

All animal experiments were performed in accordance with European and German legal and ethical regulations and approved by the responsible review boards and authorities.

*p.o. infection with scrapie brain homogenate*. Outbred Syrian hamsters were fed individual food pellets doused with 100 μl of a 10% (w/v) hamster brain homogenate (corresponding to 10 mg of tissue) from terminally ill 263K scrapie–infected donors or uninfected controls as previously described [11]. Scrapie-infected animals were humanely sacrificed by exposure to CO_2_ at 70 dpi (*n* = 5), 100 dpi (*n* = 5), and 130 dpi (*n* = 5), at the onset of at least two clinical signs for scrapie such as tremor of head or whole body, incoordination of gait, or difficulty in rising up from a supine position (*n* = 5; 138 dpi, 138 dpi, 139 dpi, 142 dpi, and 146 dpi), and at the terminal stage of disease (*n* = 5; 157 dpi, 158 dpi, 165 dpi, 169 dpi, and 171 dpi). Control animals, i.e., mock-challenged age-matched hamsters (three for each time-point), were sacrificed at corresponding time points.

*i.c. infection with scrapie brain homogenate*. Five outbred Syrian hamsters were intracerebrally infected with 50 μl aliquots of 1% (w/v) hamster brain homogenates in Tris-buffered saline ([TBS] 10 mM Tris HCl, 133 mM NaCl [pH 7.4]) from terminally ill donors challenged with 263K or BSE-H agent, or from uninfected control hamsters. The BSE-H agent was isolated in our laboratory after one passage of BSE agent from cattle in mice and subsequent transmission to hamsters [39]. The recipients (*n* = 5 for 263K, *n* = 6 for BSE-H) were humanely sacrificed at the end stage of clinical disease at the following dpi: 263K—83, 84, 87, 90, and 93 dpi; BSE-H —296, 296, 300, 310, 317, and 324 dpi.

*i.c. infection by implantation of s.w. contaminated with scrapie agent.* s.w. contaminated with 10% (w/v) brain homogenate from 263K scrapie hamsters were prepared as previously described [40] and intracerebrally implanted under anesthesia into reporter animals using a stereotaxic apparatus (K. Lemmer , M. Mielke, G. Pauli, and M. Beekes, unpublished data). Animals (*n* = 4) were humanely sacrificed at the terminal stage of scrapie at the following dpi: 91, 91, 91, and 106.

*f.p. infections with scrapie brain homogenate*. Outbred Syrian hamsters were challenged peripherally via the f.p. as described elsewhere [41] by inoculation of 50 μl of 1% (w/v) hamster brain homogenates in TBS from terminally ill 263K scrapie hamsters. Sacrification was performed humanely at the terminal stage of disease at the following dpi (*n* = 4): 117, 117, 131, and 147.

### Sample preparations.

*Preparation of hamster tissue samples for Western blot analyses.* After sacrifice, skin specimens from different regions of the body were dissected and the fur was removed by shaving. The skin specimens consisted of epidermis, dermis, and subcutis. All samples were cut into small pieces and stored at −80 °C until examination. The mass of the skin samples used for Western blot analyses ranged from approximately 40 to 100 mg. Instruments used for the preparation of samples were carefully cleaned after removal and processing of each specimen in order to avoid cross-contamination. Skin samples from the hindlimb, forelimb, head, back, and abdomen were taken from p.o.- and s.w.-infected animals. From the hamsters i.c.- or f.p.-inoculated with 263K scrapie and the animals i.c.-inoculated with BSE-H agent, skin samples were dissected from the forelimb and hindlimb.

*Preparation of hamster tissue samples for PET blot and immunohistochemical analyses.* For morphological PET blot analyses on the distribution and location of PrP^Sc^ in the skin, specimens from the head, the snout, the abdomen, and the foreleg were collected. Samples were taken from hamsters that developed terminal symptoms of scrapie after peroral or s.w. infection and from age-matched perorally mock-infected controls sacrificed at corresponding time-points. The specimens were either immediately snap frozen or fixed in paraformaldehyde (4% [w/v] in PBS) or Carnoy's solution containing 60% isopropanol, 30% dichloromethane, and 10% acetic acid for up to 48 h and embedded in paraffin. Sections were placed on glass slides and a subset was stained with haematoxylin and eosin (H&E).

*Sample preparation from sheep naturally infected with scrapie.* Skin samples from field cases of ovine scrapie were collected during autopsies of cohort animals that were eradicated after identification of an index scrapie case in the respective flock. The samples analysed in this study originated from five scrapie cases (Table 1, Sc1–Sc5) and four scrapie-free control sheep. All scrapie cases were older than 4 years as determined by their dental status and showed loss of wool due to scrubbing their coat. They also nibbled off their fleece on the legs over the carpal and tarsal joints where the straight coat becomes wooly. In all scrapie-infected animals, a marked weight loss was present. The diagnosis of scrapie was confirmed according to Office International des Epizooties standards by histopathology and by immunohistochemical detection of PrP^Sc^ in the brain stem and cerebellum. Apart from animal Sc4, PrP^Sc^ could also be detected in the tonsils from all scrapie sheep (not shown). At the polymorphic codons 136, 154, and 171 of the prion protein gene, four of them were ARQ/ARQ and one animal (Sc4) was AHQ/ARQ. According to the National Scrapie Plan for Great Britain [42], the genotypes ARQ/ARQ and AHQ/ARQ belong to risk group 3, which refers to “sheep that genetically have little resistance to scrapie and will need careful selection when used for further breeding”. It has to be noted, however, that ARQ/ARQ and AHQ/ARQ do not provide the most prevelant genotypes in scrapie-affected sheep.

**Table 1:**
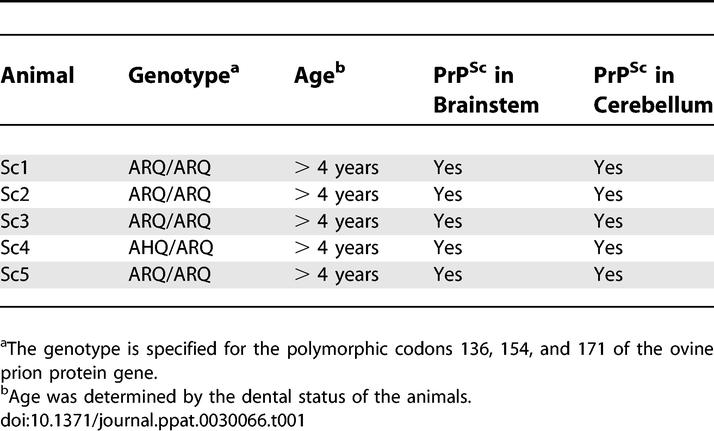
Scrapie-Infected Sheep Tested for PrP^Sc^ in the Skin

### Western blot examinations.

For Western blot analyses, samples were washed three times in TBS (10 mM Tris HCl, 133 mM NaCl [pH 7.4]) and incubated in a rocking device at 37 °C for 4 h in 900 μl of TBS containing 2 mM CaCl_2_ and 0.25% (w/v) collagenase A (Roche, http://www.roche.com). For positive controls, skin tissue from orally mock-infected control donors was spiked by adding 5 μl of a 0.1 % (w/v) 263K scrapie hamster brain homogenate (i.e., 5 μg of brain tissue) from i.c.-infected donors containing approximately 0.5 ng of PrP^Sc^ [3] prior to collagenase digestion. After ultrasonification to disrupt remaining tissue structures, the samples were centrifuged for 3 min at 500*g.* The supernatant was carefully transferred to a new cup, whereas the pellet consisting of cell debris and the rest of the fur was removed. Subsequently, PrP^Sc^ was extracted in the form of PrP27–30 from the tissue homogenates following a previously published protocol [23]. Proteinase K–digested homogenate from 263K scrapie hamster brains, used as a PrP27–30 reference in the Western blotting analyses, was prepared as outlined previously [23]. For deglycosylation [23], extracted pellets were dissolved in 20 μl of a. bidest and one fourth of the aliquots was digested using PNGase F (New England Biolabs, http://www.neb.com) according to the instructions of the manufacturer prior to Western blotting. Sodium dodecyl sulfate-polyacrylamide gel electrophoresis (SDS-PAGE) and Western blot analyses of samples from hamsters were performed as described elsewhere [23]. Western blot testing for PrP^Sc^ in skin samples from scrapie-infected sheep was similarly performed using the primary antibodies ICSM-18 and P4 to label the ovine prion protein. PrP signals were visualized on an X-OMAT AR (Kodak, http://www.kodak.com) film. Films were exposed for 5–30 min.

### PET blot examinations.

The PET blot detection of proteinase K–resistant PrP deposits in sections of skin samples was performed on paraformaldehyde- and Carnoy-fixed specimens. For PET blot examinations of skin tissue, modifications of the original protocol [43] were necessary to remove connective tissue. After prewetting blots with TBST (10 mM TrisHCl [pH 7.8], 100 mM NaCl, 0.05 % [w/v] Tween 20), sections were digested with 1.5 mg/ml collagenase A (Roche) in a buffer containing 10 mM TrisHCl (pH 7.8), 100 mM NaCl, 100 mM CaCl_2_, and 0.1% (w/v) Brij 35 for 30 min at 60 °C, followed by digestion using 250 μg/ml proteinase K (Roche) in PK digestion buffer (10 mM TrisHCl [pH 7.8], 100 mM NaCl, 0.1% Brij 35) for 8 h at 55 °C. After this step, the membrane-attached proteins were fixated to the membrane. The proteins on the membranes were denatured with 3 M guanidine isothiocyanate in 10 mM TrisHCl (pH 7.8) for 20 min. Immunodetection was performed after preincubation in blocking solution (0.2% [w/v] casein in TBST) for 30 min. The monoclonal antibody 3F4 ([44]; diluted 1:3,000) was used as primary antibody and an alkaline phosphatase-coupled rabbit anti-mouse antibody (Dako, http://www.dako.com) at a dilution of 1:500 as secondary antibody. Visualization of antibody binding was achieved by using NBT/BCIP. Blots were examined using an Olympus dissecting microscope.

### Immunohistochemical examinations.

Frozen tissue sections as well as Carnoy-fixed sections of about 4 μm were postfixed in paraformaldehyde (4% [w/v] in PBS) for 1 h and washed in tap water to avoid pigment deposition. Antigen retrieval was done using 4 M guanidine hydrochloride for 25 min and by microwaving five times for 3 min at 700 watts in 1 M citric acid at pH 6.0. After blocking with 0.2% casein in TBST, the primary antibody 3F4 (diluted 1:200) was used for PrP detection. Antibodies against S-100 protein (rabbit polyclonal, 1:200 in PBS; Dako,) and neurofilament (SMI31 mouse monoclonal IgG1, 1:20,000 in PBS; Sternberger Monoclonals, http://www.crpinc.com) were applied to detect small nerve fibres. As secondary antibody, a HRP-conjugated goat anti-mouse IgG1 antibody (1:400 in PBS; Dianova, http://www.dianova.de) was used for detecting the antibody SMI31. The S-100 antibody was detected using a polyvalent HRP-conjugated goat anti-rabbit antibody (EnVision, Dako). For 3F4 doublestaining, we used a biotinylated goat anti-mouse IgG2a antibody (1:400 in PBS; Dianova) or a goat anti-mouse IgG antibody without restriction to Ig-subclasses (1:100; Dako). Visualization of the different epitopes was performed using Cy2-conjugated streptavidine (green fluorescence, 1:500; Dianova) and a Cy3-conjugated goat anti-HRP antibody (red fluorescence, 1:100; Dianova). Slides were examined on an Olympus fluorescent microscope using analysis software, and Adobe Photoshop software was used for picture processing.

## Abbreviations

BSE: bovine spongiform encephalopathy
BSE-H: hamster-adapted BSE
CWD: chronic wasting disease
dpi: days post infection
f.p.: foot pad
H&E: haematoxylin and eosin
i.c.: intracerebral
PET: paraffin-embedded tissue
p.o.: oral
PrP: prion protein
PrP27–30: protease-resistant core of the pathological prion protein PrP^Sc^
PrP^C^: cellular isoform of the prion protein
PrP^Sc^: pathological isoform of the prion protein
s.w.: steel wires
TSE: transmissible spongiform encephalopathy
vCJD: variant Creutzfeldt-Jakob disease
